# Multimodal widefield fluorescence imaging with nonlinear optical microscopy workflow for noninvasive oral epithelial neoplasia detection: a preclinical study

**DOI:** 10.1117/1.JBO.25.11.116008

**Published:** 2020-11-16

**Authors:** Rahul Pal, Paula Villarreal, Xiaoying Yu, Suimin Qiu, Gracie Vargas

**Affiliations:** a Massachusetts General Hospital and Harvard Medical School, Athinoula A. Martinos Center for Biomedical Imaging, Charlestown, Massachusetts, United States; b The University of Texas Medical Branch, Biomedical Engineering and Imaging Sciences Group, Galveston, Texas, United States; c The University of Texas Medical Branch, Department of Neuroscience, Cell Biology, and Anatomy, Galveston, Texas, United States; d The University of Texas Medical Branch, Department of Preventive Medicine and Population Health, Galveston, Texas, United States; e The University of Texas Medical Branch, Department of Pathology, Galveston, Texas, United States

**Keywords:** multimodal imaging, widefield fluorescence, nonlinear optical microscopy, oral cancer, inflammation, cancer detection

## Abstract

**Significance:** Early detection of epithelial cancers and precancers/neoplasia in the presence of benign lesions is challenging due to the lack of robust *in vivo* imaging and biopsy guidance techniques. Label-free nonlinear optical microscopy (NLOM) has shown promise for optical biopsy through the detection of cellular and extracellular signatures of neoplasia. Although *in vivo* microscopy techniques continue to be developed, the surface area imaged in microscopy is limited by the field of view. FDA-approved widefield fluorescence (WF) imaging systems that capture autofluorescence signatures of neoplasia provide molecular information at large fields of view, which may complement the cytologic and architectural information provided by NLOM.

**Aim:** A multimodal imaging approach with high-sensitivity WF and high-resolution NLOM was investigated to identify and distinguish image-based features of neoplasia from normal and benign lesions.

**Approach:** *In vivo* label-free WF imaging and NLOM was performed in preclinical hamster models of oral neoplasia and inflammation. Analyses of WF imaging, NLOM imaging, and dual modality (WF combined with NLOM) were performed.

**Results:** WF imaging showed increased red-to-green autofluorescence ratio in neoplasia compared to inflammation and normal oral mucosa (p<0.01). *In vivo* assessment of the mucosal tissue with NLOM revealed subsurface cytologic (nuclear pleomorphism) and architectural (remodeling of extracellular matrix) atypia in histologically confirmed neoplastic tissue, which were not observed in inflammation or normal mucosa. Univariate and multivariate statistical analysis of macroscopic and microscopic image-based features indicated improved performance (94% sensitivity and 97% specificity) of a multiscale approach over WF alone, even in the presence of benign lesions (inflammation), a common confounding factor in diagnostics.

**Conclusions:** A multimodal imaging approach integrating strengths from WF and NLOM may be beneficial in identifying oral neoplasia. Our study could guide future studies on human oral neoplasia to further evaluate merits and limitations of multimodal workflows and inform the development of multiscale clinical imaging systems.

## 1 Introduction

Oral squamous cell carcinoma (OSCC) accounts for almost 94% of all oral cancers with ∼53,000 new diagnoses every year solely in the United States (US).^1^ The five-year survival rate of patients with advanced OSCC involving regional lymph node invasion or beyond is ∼47% (regional) and 20% (distant) but can be as high as 84% when cancers are diagnosed and treated when detected in the localized primary site.^2^ It is estimated that the percent of cases diagnosed at advanced stages, defined as regional or distant cancers, range from 64 to beyond 70%, motivating approaches that facilitate early detection and intervention.^3^^,^^4^ The most comprehensive US source is the SEER Cancer Statistics Review (CSR) 1975–2016, which indicates 31% of cases are diagnosed at early stage (local), 64% at advanced stages (regional or distant), and 5% at unknown stages.^5^ OSCCs, primarily oral epithelial dysplasia (OED), have potential to develop from neoplasia, an abnormal growth of the epithelium due to a malignant or a benign precancerous lesion.^6^^,^^7^ With grade of OEDs remaining the key factor to assess risk for transformation of such lesions, there have been increased efforts to detect OEDs harbored in lesions to aid in clinical decisions.^8^^,^^9^ There remains a need for methods that more effectively support detection efforts.

The current clinical approach for detection of OSCC/OED begins with conventional oral examination (COE) comprising inspection of the tissue surface with white light visualization combined with palpation of the oral cavity to guide biopsy, performed by an experienced physician.^10^ COE is usually performed by a dentist, an otolaryngologist (most commonly), or a community practitioner (less commonly), on lesions identified by patients or found during routine exam.^11^^–^^13^ Differentiating between benign and high-risk lesions by COE is a noted challenge. Accurate histopathologic diagnosis relies on a number of critical steps: evaluation of COE provided by an experienced head and neck and oral and maxillofacial surgeon, who are most likely to recognize lesions with malignant potential compared to dentists and general practitioners,^14^ biopsy site, and a pathologist’s interpretation of results. Benign conditions, such as inflammation, often present similar visual cues to OSCC/OED leading to false positives and over sampling during biopsy. A recent meta-analysis reported high sensitivity (90%) and poor specificity (31%) of COE for high-risk precancerous lesions (OEDs), noting COE cannot reliably distinguish these from benign lesions.^15^ Noninvasive methods to identify signs of oral neoplasia, including OED, are needed to help clinicians identify suspicious lesions with higher specificity and for biopsy guidance.^16^

Because optical imaging offers a number of contrast mechanisms for surveying tissue biochemical and structural characteristics over a range of resolutions and sampling areas, a variety of optical methods have been proposed for detection of neoplasia in the oral cavity. These include large area (widefield) methods that provide topical/surface assessment as well as high-resolution methods in some cases with depth imaging. Detection of surface anomalies in large tissue areas by autofluorescence imaging has shown promise in improving contrast between normal and neoplastic tissue.^17^^–^^19^ Widefield fluorescence (WF) imaging complements COE by providing information on fluorescence properties from tissue surfaces at a scale similar to COE (tens of centimeters) making it an easily adaptable approach in clinical settings. Imaging relies on alterations of endogenous fluorescence in neoplasia using a blue or ultraviolet light excitation (360 to 460 nm) with images assessed visually or captured by a digital camera.^18^^,^^20^ Contrast for neoplastic tissue is obtained by loss of blue-green autofluorescence. Loss of fluorescence is expected to arise from a variety of sources, such as loss of collagen in the extracellular matrix (ECM), and increased thickness as well as scattering of epithelium among other factors.^21^ Increased red autofluorescence may also occur and is generally attributed to protoporphyrin IX.^22^ Although WF imaging shows promise in detection and monitoring of OSCC/OED resulting in detection sensitivities in the range of 50% to 90%, specificity for neoplasia is significantly lower, as low as 30% to 40%, as shown in clinical studies.^23^^,^^24^ Clinical WF, capturing blue-green autofluorescence with 430-nm excitation, has been suggested to improve detection of neoplastic oral lesions versus COE alone.^25^^,^^26^ However, inclusion of benign conditions (e.g., keratosis and inflammation) in study populations results in lower specificity, to as low as 15% to 30%.^27^^–^^33^ Studies suggest improvements by use of multichannel red-to-green (RG) fluorescence intensity ratio over individual fluorescence channels (red or green).^33^^–^^35^ Thus far, the use of WF imaging as a stand-alone technology has been limited even with FDA-approved commercial systems available, and it is of interest to investigate their use as part of multimodal efforts that take advantage of large area assessment and somewhat high sensitivity for oral neoplasia detection.

High-resolution optical imaging methods, such as confocal reflectance microscopy (CRM), optical coherence tomography (OCT), and nonlinear optical microscopy (NLOM), such as multiphoton microscopy (MPM) and second harmonic generation microscopy (SHGM), have been investigated for detection of oral epithelial neoplasia.^36^^,^^37^ These methods provide depth resolved microstructural information at high spatial resolution with neoplasia. Preclinical studies have shown promise and stimulated research for clinical translation.^38^^,^^39^ CRM and OCT are based on scattering of light in tissue and do not contain molecular information.^40^ Although CRM has limited imaging depth (tens of micrometers), OCT provides limited resolution and contrast that is not ideal for subsurface cellular imaging. Normal human oral mucosa has a mean epithelial thickness of roughly 330  μm and may expand during neoplastic development.^41^ Moreover, since oral neoplasia originates in the epithelium, primarily in the basal cell layer, the imaging depth provided by CRM and the resolution provided by OCT may not be sufficient for identifying early signs of neoplasia in the human oral mucosa. NLOM informs on both microstructure and molecular signatures of neoplasia such as by MPM imaging based on endogenous fluorescence from cytoplasmic NADH and FAD.^42^^–^^45^ NLOM provides deep tissue (hundreds of micrometers) imaging, optical sectioning capability for *in vivo* three-dimensional imaging, and high lateral resolution (∼300  nm). High imaging depth of NLOM is particularly important as an *in vivo* deep tissue imaging technology would allow identification of early signs of neoplasia. Several groups have previously evaluated methods of NLOM, such as MPM and SHGM, for label-free detection of OSCC/OED in preclinical animal models^36^^,^^37^^,^^43^^–^^50^ and *ex vivo* human tissue^49^^,^^50^ with one study exploring *in vivo* harmonic generation imaging in normal oral cavity. Imaging by MPM reveals neoplastic features such as increased thickness of epithelium, nuclear and cellular atypia, and increased nuclear to cytoplasmic ratio, whereas SHGM informs about degradation and remodeling of collagen in the ECM during neoplastic transformation.^36^^,^^37^^,^^42^^–^^48^ In this preclinical study of the mucosal surface in a hamster model of neoplasia, high sensitivity and specificity for OED were achieved, even in the presence of benign conditions (inflammation), with MPM-based features that are routinely used in histopathological grading as well as unique features such as the shape of the epithelial-connective tissue interface (ECTI).^36^ As contrast for MPM relies on metabolic cofactors NADH and FAD, image-based methods to evaluate metabolic activity of normal and neoplastic tissue have been developed and shown promise in cell culture and preclinical studies.^45^^,^^51^ These recent advances in the field have established NLOM as a potential method for prebiopsy lesion identification in the oral cavity, and feasibility of miniaturization into fibered handheld instruments amenable to the oral cavity has been established paving routes for potential clinical implementation.^52^^–^^54^ However, NLOM is inherently limited by the small scanning field of view (hundreds of micrometers), whereas investigation of oral neoplasia requires a large area assessment (tens of centimeters).

The need for *in vivo* detection of neoplasia to identify OEDs has led to the development of several approaches with a focus on multimodal imaging. In a multimodal imaging approach, two or more imaging modalities that could be used simultaneously or sequentially with capabilities to provide complementary information are employed.^55^^,^^56^ Current efforts in development for multimodal *in vivo* imaging systems include fluorescence lifetime microscopy (FLIM)^57^ and CRM,^58^ OCT and fluorescence imaging,^59^ FLIM and OCT,^60^ macroscopic white-light, autofluorescence, and high-resolution microendoscopy (HRME) using proflavine dye.^56^ To the authors’ knowledge, no known study has examined the combination of widefield autofluorescence imaging with NLOM in the oral cavity.

Thus in this study, we evaluated a new multimodal approach combining macroscopic WF imaging and microscopic depth imaging by NLOM for detection of oral neoplasia, with assessments in a preclinical hamster cheek (buccal) pouch model of OSCC/OED. This approach takes advantage of high sensitivity of WF imaging and high-resolution, deep tissue imaging capability of NLOM for high-specificity detection of oral neoplasia. A second differentiator from most past studies that evaluate optical detection of epithelial cancer is that inflammation, a critical confounding factor in OSCC/OED detection, is included in the presented studies. Using univariate analyses and multivariable statistical models, we demonstrate improved performance of the multimodal approach over WF alone, especially in the presence of inflammation.

## 2 Materials and Methods

### 2.1 Animal Model

Animal studies conformed to the Guide for the Care and Use of Laboratory Animals and were approved by the Institutional Animal Care and Use Committee at the University of Texas Medical Branch. Male Golden Syrian Hamsters (Harlan Laboratories) were treated, starting at age four weeks, thrice weekly for 8 to 12 weeks with topical 0.5% 9,10-dimethyl-1,2-benzanthracene (DMBA) in mineral oil on the left buccal pouch using a camel hair paintbrush (width 1/400) to induce neoplasia (n=25).^61^ This model manifests histological and molecular similarities to human OED and OSCC^61^^,^^62^ and at 8 to 12 weeks presents with heterogeneous oral mucosa with sites of inflammation, leukoplakia with OED, and small exophytic tumors across the surface. Age-matched control animals (n=7) were treated with only mineral oil following the same schedule. In another group, a solution of 1.4% sodium lauryl sulfate (SLS), 29% calcium pyrophosphate, and 18% glycerol in sterilized water was applied topically to the buccal pouch in the same manner for four consecutive days to induce inflammation (n=5) with imaging on day 5.^63^^,^^64^ Animals were anesthetized with an intraperitoneal injection of 100-mg/kg ketamine and 2.5-mg/kg xylazine for imaging, and the buccal pouch was gently pulled and stretched flat onto a sample holder using pins and rinsed with PBS.

### 2.2 Imaging

**Fig. 1:**
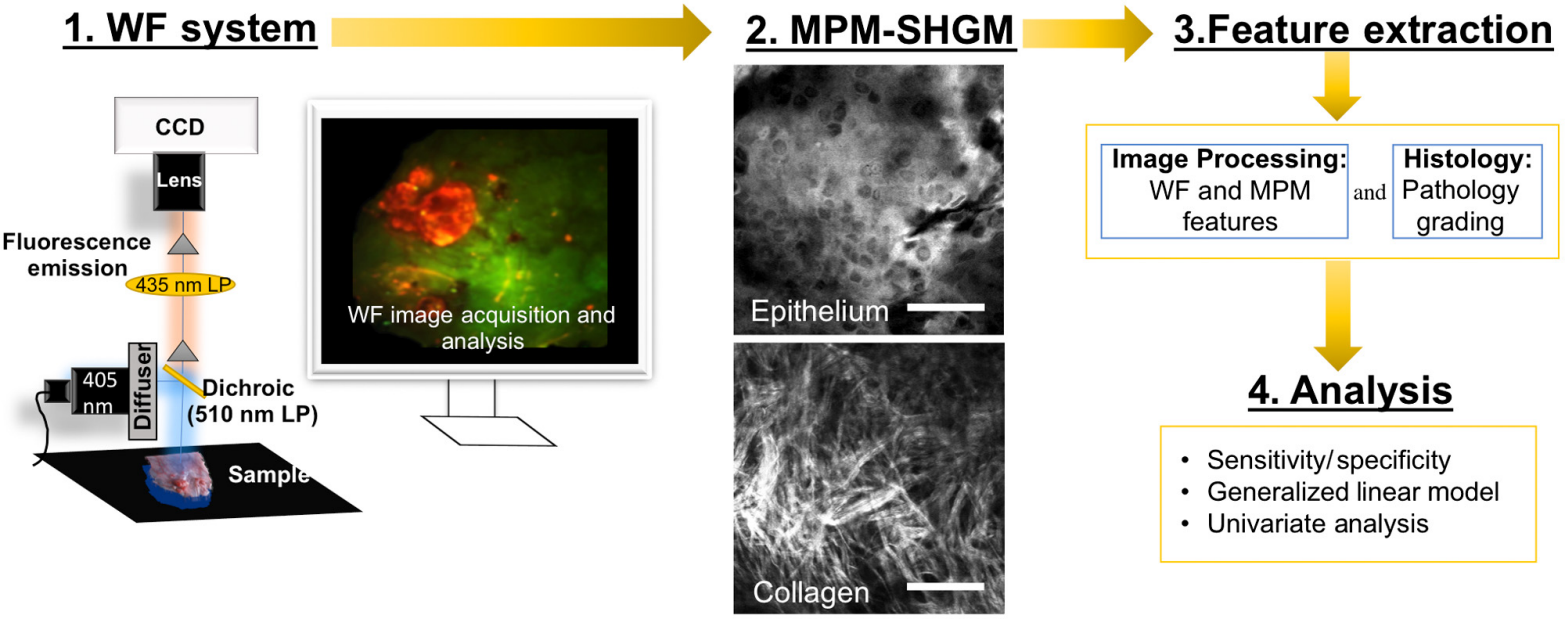
Multiscale imaging workflow. In part 1, WF imaging was assessed for sensitivity and specificity to detect oral epithelial neoplasia based on RG fluorescence. In part 2, sequential imaging was performed with WF used to identify sites for *in vivo* NLOM (MPM-SHGM) microstructural assessment (step 2). After imaging is completed, normalized RG values and microstructural features are extracted (step 3) and statistical analysis is performed (step 4) to categorize sites as normal/benign or neoplastic. The WF system consisted of a 405-nm high-power LED, a dichroic 510-nm LP filter, an emission filter 435-nm LP, and a Nikon digital Ds-Fi1 camera with a 60-mm 1:2.8 lens. Scale bar: 100  μm.

The multimodal imaging workflow used is outlined in Fig. 1. Part 1 employed imaging with a large area WF imaging system alone to characterize performance for detection of neoplasia on the buccal pouch. Part 2 followed a sequential workflow of WF followed by NLOM *in vivo*. Image-based feature extraction and statistical analysis of features are described below.

#### 2.2.1 WF imaging system

The WF imaging system (Fig. 1) used a collimated 405 nm (full-width half-max = 10 nm) LED light source (M405L3-C1, ThorLabs, Newton) for excitation of autofluorescence. The collimated excitation beam was steered onto the cheek pouch mucosa for *in vivo* imaging through a dichroic filter (510-nm longpass, LP) to collect all green (collagen, redox coenzymes) and red (porphyrin) autofluorescence. The 435-nm long-pass filter was placed as an extra measure to eliminate any blue illumination or emission leakage through the dichroic. Irradiance at the imaging field of view was kept constant at $2.1\;\mathrm{mW}/{\mathrm{cm}}^{2}$ throughout the study. Fluorescence images were captured with a Nikon DS Fi1 color camera with an AF Micro NIKKOR 60 mm 1:2.8 lens. Images were captured as 24-bit RGB tiff using Nikon Elements software (Nikon DS-U2 Ver4.60). Red and green fluorescence channels with FWHM of 580 to 700 nm and 500 to 580 nm, respectively, were separated postimaging using the camera Bayer mask. Spectral overlap between the red and green channels of the camera occurs at the spectral tail ends and spanned from 560 to 600 nm. An ∼5-cm diameter uniform illumination field was achieved, sufficient to image the buccal mucosa. Resolution of the WF system was tested using a US Air Force (USAF) resolution target—a line spacing of 24.8  μm in group 4 and element 3 was discriminated.

#### 2.2.2 *In vivo* WF imaging

For WF imaging, hamsters were positioned to capture tissue autofluorescence images of the entire buccal pouch (field of view = 5 cm diameter). Exposure and gain settings were established through pilot images and for each imaging session, a set of images comprising exposure and gain settings within this range were obtained. Part of the sample holder served as an internal control for variation in illumination and used to normalize intensity. White light images of the pouch were also captured to localize and record sites for NLOM and biopsy.

#### 2.2.3 NLOM

NLOM composed of MPM and SHGM was performed using a custom-built NLOM system.^36^ The system used a $\mathrm{Nd}:{\mathrm{YVO}}_{4}$-pumped Ti:sapphire femtosecond (∼100  fs, 82 MHz) pulsed laser source (Tsunami, Spectra Physics), tunable over 750 to 1000 nm. For MPM, two-photon excitation was achieved with 780 nm and autofluorescence collected through a 450-to 650-nm broadband emission filter, and 840 nm was used for second harmonic generation (SHG) collected through a 420/20-nm bandpass filter. An average excitation power of 28 mW was used at the tissue. A long working distance 10×, 0.3 NA air objective (Plan-Neofluar) was used for localization of regions of interest and a 40×, 1.2 NA, C-Apochromat, water immersion objective was used for microscopy, with field of view of 320×320  μm and working distance of 180  μm. Three-dimensional microscopy was performed by obtaining z stacks using 1  μm z steps and 8-bit images with 512×512  pixels and 0.625  μm pixel size. NLOM was performed following full buccal pouch WF imaging. Sites chosen for NLOM imaging included sites identified to have increased red autofluorescence and/or decreased green autofluorescence. Additional sites lacking alterations in red and/or green autofluorescence compared to the background or the reference were also chosen througout the surface. Multiple areas from each mucosal tissue were selected for NLOM imaging, with obvious tumors excluded because they protruded above the surface and with flattening often resulted in bleeding. Sites excluded from WF analysis as specified below were also excluded from NLOM.

### 2.3 Histopathology

H&E stained sections from biopsied sites (all those imaged *in vivo* by WF alone (part 1) or WF/NLOM (part 2) were imaged with an Olympus IX71 inverted brightfield microscope using a 20×, 0.75 NA air objective. Sections were graded into multiple pathologic categories (normal, inflammation, mild OED, moderate OED, severe OED, and OSCC) with the most central region having the most severe pathological features assigned to each site. Grading was performed according to the World Health Organization criteria for architectural and cytological changes in neoplastic epithelium.^65^

### 2.4 Image Processing and Data Analysis

Two sets of analyses were carried out: (1) analysis of buccal WF images to determine sensitivity and specificity for detecting neoplasia as a stand-alone method and for comparison with previous WF studies (part 1) and (2) analysis of *in vivo* multimodal imaging data from WF followed by NLOM to evaluate combined imaging performance (part 2). Part 2 composed of (a) receiver operator characteristic (ROC) curve analysis of NLOM performed after WF (part 2a) and (b) a generalized linear model (GLM) analysis that examined WF and NLOM metrics together for a combined approach sensitivity/specificity assessment (part 2b).

### 2.5 WF Image Analysis (Normalized RG)

WF images were analyzed using FIJI (Image J, NIH Image) to calculate the normalized red-to-green intensity ratio (normalized RG) with separation of red and green channels form the camera Bayer mask. Analysis of red and green autofluorescence was by sampling sites across the buccal pouch. Sites included for analysis were regions having increased red emission and reduced green autofluorescence relative to surrounding areas and the reference standard. Sites that did not have increased red or decreased green (basal level) were also chosen. These included visually indistinct sites as well as those having surface roughness and abnormal coloring in white light. Sites near edges of the pouch or having injury (e.g., due to pins) and sites with visible folds were excluded from analysis. Although some tumors visible to the eye were included in WF analysis, tumors contaminated by blood (advanced tumors that bled easily) were excluded from all analyses.

Based on these criteria, 3 to 5 sites were selected from each buccal mucosa providing 112 sites (37 control, 16 inflammation, 28 OED, and 31 OSCC). Three regions of interests (ROIs) of size 20×20  pixels were created within each site and the average intensity in each ROI for red ($I_{R1}$, $I_{R2}$, and $I_{R3}$) and green ($I_{G1}$, $I_{G2}$, and $I_{G3}$) channels were measured. A ratio of RG intensities for each ROI ($I_{\mathrm{RG}1}$, $I_{\mathrm{RG}2}$, and $I_{\mathrm{RG}3}$) was calculated. RG intensity ratios were measured ($I_{C\text{-}\mathrm{RG}1},I_{C\text{-}\mathrm{RG}2},\ldots ,I_{C\text{-}\mathrm{RG}5}$) from five ROIs on the internal control (sample holder) found on each buccal pouch image. An average RG intensity ratio ($I_{C\text{-}\mathrm{RG}}=\overset{5}{\underset{i=1}{\sum }}I_{C\text{-}\mathrm{RG}i}/5$) of the internal control was used as a normalization factor for RG intensity ratios ($I_{\mathrm{RG}1}$, $I_{\mathrm{RG}2}$, and $I_{\mathrm{RG}3}$) from sites.^66^ This normalization was performed by individually dividing $I_{\mathrm{RG}1}$, $I_{\mathrm{RG}2}$, and $I_{\mathrm{RG}3}$ by the average RG intensity ratio of the internal control ($I_{C\text{-}\mathrm{RG}}$). The normalized RG intensity ratios for three ROIs from each site were averaged to obtain the normalized RG value ($I_{\mathrm{RG},\text{norm}}$).

### 2.6 MPM-Based Cytology

In part 2, 33 normal, 11 inflammation, and 19 OED sites were imaged by NLOM following WF. MPM-SHGM stacks were evaluated in FIJI (Image J and NIH Image) to extract quantitative measures representing cellular atypia (crowding, nuclear enlargement, and nuclear pleomorphism). Quantitative measurements were made for nuclear area at the basal epithelium and the coefficient of variance of nuclear area to represent anisonucleosis/nuclear pleomorphism. These were quantified for three separate image planes 5 to 10  μm apart in the basal layer in each image stack. Outlines of 20 nuclei from each image plane were manually delineated and the nuclear areas were calculated by measuring the lengths of major and minor axes of each nucleus. The shape of nuclei of these epithelial cells was treated as ellipsoidal and the area of each nucleus (∼20 nuclei from each image plane and ∼60 nuclei from each image stack) was determined using the general formula for area of an ellipse. Coefficient of variance in nuclear area (CoVa) is represented as ratio of standard deviation over mean nuclear area.

### 2.7 Delineation of Epithelial-Connective Tissue Interface

3D MPM/SHGM volumetric images were processed using FIJI and Imaris (Bitplane, Version 7.4.2) to extract ECTI shape parameters.^36^ The ECTI is defined as the junction between epithelium and ECM at the basement membrane, visualized at the interface between MPM fluorescence and ECM SHGM. In neoplasia, hyperproliferation of the epithelium combined with ECM remodeling results in focal epithelial thickening and altered shape of the ECTI, which in buccal mucosa transforms from a flat “sheet” to deformed surface.^36^^,^^67^^,^^68^ “ECTI contour,” defined as the surface area of ECTI with respect to a flat surface was used as an ECTI shape parameter, found using Heron’s approximation for a curved surface^36^^,^^69^ It was previously found to correlate highly with neoplasia since regions of deformed ECTI are found below regions of cellular atypia.^36^

### 2.8 Statistical Analysis

Statistical comparisons between groups for individual modality (WF or NLOM) measures were performed by single factor ANOVA with Tukey’s *post hoc* test and p<0.05 is considered significant (in figures, p<0.05 is represented by single asterisk “*” and p<0.01 by double asterisk “**”). ROC curves were generated using SAS software (SAS Institute Inc.) to calculate area under the curve (AUC), sensitivity, and specificity. Combined modality statistics were also performed using GLM in the statistical software R (version 3.2.3). For the GLM, one WF image parameter (normalized RG) and two best performing NLOM parameters based on univariate analyses (ECTI contour and basal nuclear CoVa) were used in pairs as input response variables. A GLM is a variation of linear regression that accounts for response variables that do not show a normal distribution.^70^ GLM may be applied when the response variable is categorical, having two possible outcomes (in this case neoplastic and non-neoplastic). Thus two-class GLMs for three pairs of input response variables (RG-ECTI contour and RG-CoVa) were created and GLM-assigned coefficients for each pair were obtained from models. Coefficients were used to create a binary classification of *in vivo* imaged areas: neoplastic and non-neoplastic (normal and inflammation). Sensitivity and specificity of the GLMs were obtained by generating ROC curves comparing GLM-based binary classification with the gold standard histology-based classification as the outcome. Additionally, a multivariable model was generated with all three input response variables combining WF and NLOM parameters together (normalized RG, basal nuclear CoVa, and ECTI contour). This was done using a forward selection that started with normalized RG and continued with addition of one NLOM parameter in each cycle until the largest separation between neoplastic and non-neoplastic tissue was found.

## 3 Results

### 3.1 *In vivo* WF Imaging for Large Scale Surveillance

**Fig. 2:**
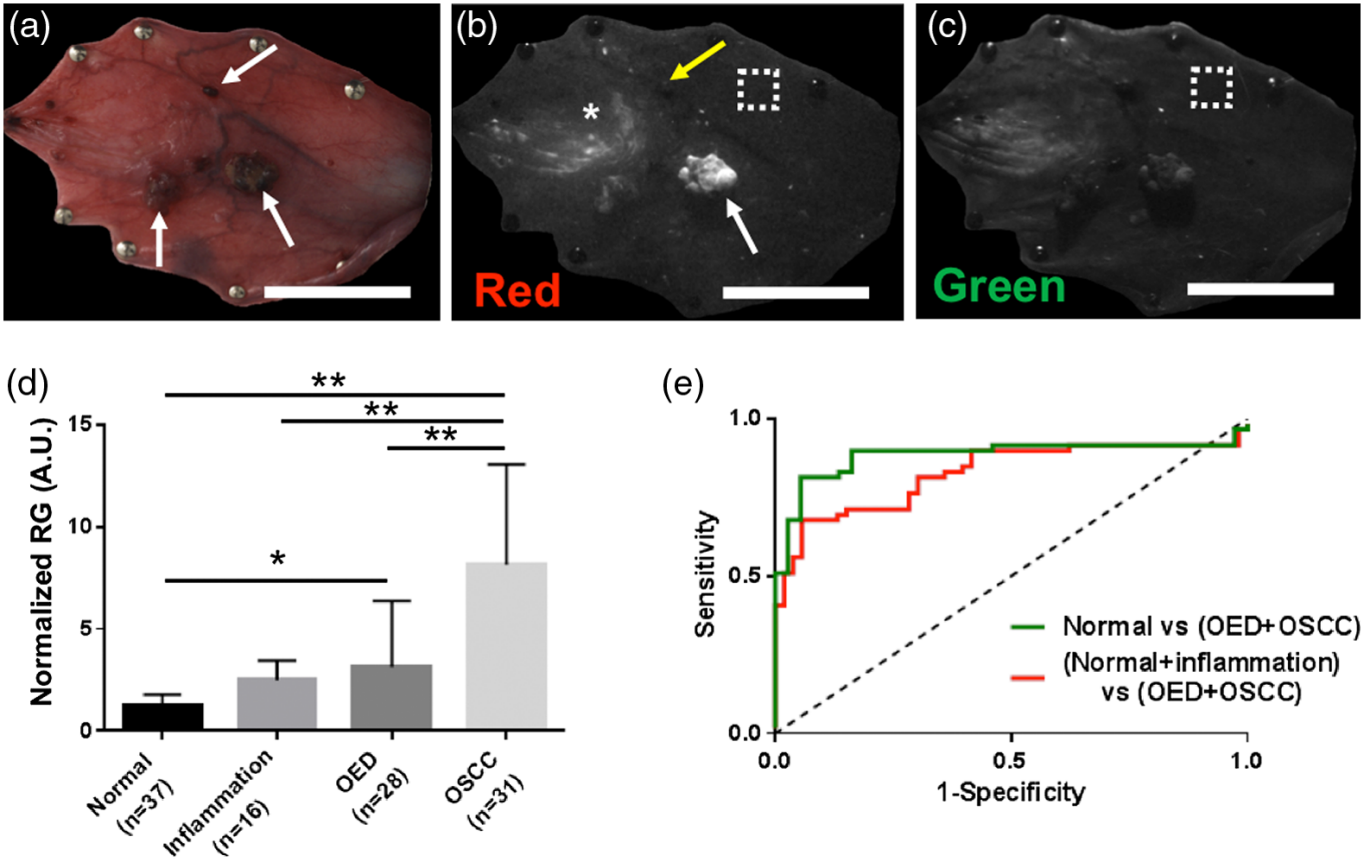
Large area *in vivo* WF imaging: representative white light and WF images of a DMBA-treated hamster cheek pouch are shown: (a) white light image; (b) autofluorescence in the red channel; and (c) autofluorescence in the green channel. Examples of OSCC (1a, “arrows”), dysplasia (1b, “*”), a necrotic tumor (1b, “yellow arrow”), a normal area (1b and 1c, dotted white box) are outlined; (d) quantitative normalized RG values for all groups of normal, inflammation, OED, and OSCC (study 1a). Numbers in brackets indicate number of sites analyzed in each group; (e) ROC curves of normal versus neoplasia (green line) and (normal + inflammation) versus neoplasia (red line). The neoplasia group combines OED and OSCC groups. “*” p<0.05; “**” p<0.01 AUC=0.88 at sensitivity=89.83%, specificity=83.78% in the case of normal versus neoplasia (green curve) inclusion of inflammation resulted in lowered specificity=64.15%, similar sensitivity (83.05%), and AUC=0.83 in the case of benign versus neoplasia (red curve). Scale bar: 2 mm.

WF imaging following white light examination in the buccal pouch model highlighted autofluorescence characteristics of tumors and areas surrounding tumors. Preliminary visual examination identified several tumors (arrows) protruding from the mucosal surface [Fig. 2(a)], which displayed strong autofluorescence in the red channel [Fig. 2(b), white arrow]. Necrotic tumors appeared as non-fluorescent dark regions [Fig. 2(b), yellow arrow]. The green channel [Fig. 2(c)] in general showed low autofluorescence in neoplastic regions, consistent with the literature. In addition to large non-necrotic tumors, select areas adjacent to tumors showed increased fluorescence signal in the red channel [Fig. 2(b), marked “*”]. Biopsies of such areas revealed OED by histopathology, despite lacking obvious surface abnormalities of roughness or discoloration [Fig. 2(a)]. There were also areas without surface abnormalities with no observable variation in fluorescence relative to basal values [an example is shown in Figs. 2(b) and 2(c); dotted white box]. Such areas had normal epithelial pathology.

Normalized RG values were obtained for individual ROIs of normal, inflammation, OED and OSCC, and averaged per group [Fig. 2(d)]. Average normalized RG for OSCC (8.15±4.9) was significantly higher (p<0.01) than all three non-OSCC groups (normal: 1.23±0.55, inflammation: 2.48±0.96, OED: 3.13±3.23), consistent with raw fluorescence images where OSCC showed strongest autofluorescence compared with normal, inflammation, and OED tissues in the red channel. Normalized RG for OEDs were also significantly higher than normal tissue. However, there was no significant difference between RG values in OED and inflammation or OED and OSCC. Average normalized RG for inflammation trended above but was not statistically different from normal. ROC curves in Fig. 2(e) indicate sensitivity/specificity of RG intensity analysis. Inclusion of inflammation in the normal tissue group (red curve) reduced performance drastically from when only normal tissue (green curve) was compared against neoplasia (combined OED and OSCC). ROC curves discriminated normal and neoplasia (OED + OSCC) with high sensitivity (89.83%), specificity (83.78%), and AUC (0.88), whereas inclusion of inflammation in the normal tissue group reduced sensitivity (83.05), specificity (64.15), and AUC (0.83). The sharp drop in specificity (Table 1) in the presence of inflammation resulted in lowered performance of WF imaging, with specificity being the most variable. In an analysis of a subset (n=37 [normal], 16 [inflammation], 28 [OED]), which excluded the tumors, the AUC became 0.73 (sensitivity 78% and specificity 40%) and 0.64 (sensitivity 76% and specificity 30%), without and with inflammation, respectively.

**Table 1:** Results from ROC curve analysis: AUC, sensitivity, and specificity of WF imaging

	AUC	Sensitivity	Specificity
Normal versus (OED + OSCC)	0.88	89.83	83.78
(Normal + inflammation) versus (OED + OSCC)	0.83	83.05	64.15

**Fig. 3:**
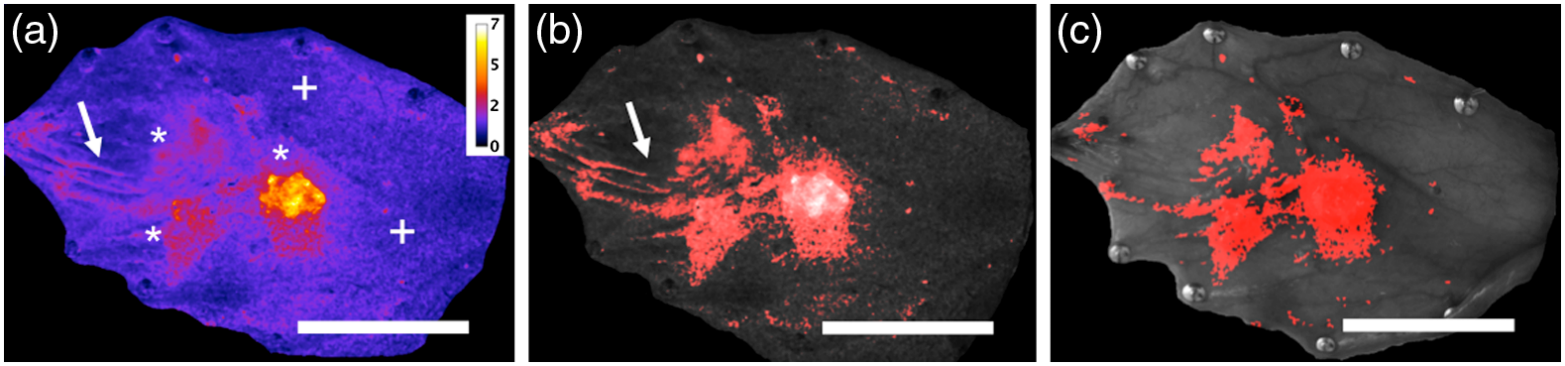
WF image processing and visualization. (a) Normalized RG map of the whole buccal pouch from Fig. 2 with a scale shown for values from 0 (dark blue) to 7 (white); (b) RG threshold map: normalized RG values above 1.46 (as obtained from ROC curve analysis) are represented in color while gray pixels represent normalized RG values less than 1.46 in this fluorescence image; and (c) locations of areas with normalized RG values >1.46 suspicious for neoplasia are overlaid on a white light grayscale photograph of the buccal mucosa. This visual map demonstrates an example visual that could be created for rapid identification of suspicious areas. Scale bar: 2 mm.

A threshold 1.46 for normalized RG value was obtained from ROC analysis for discriminating normal and neoplastic (OED + OSCC) tissue, used to create a quantitative visual map, which could be helpful in quickly spotting areas of suspicion for neoplasia. Figure 3(a) shows a normalized RG map of the entire oral mucosa of Fig. 2 with normalized RG values shown in the color scale (values between 0 and 7). Tumors and OED sites (asterisks, “*”) show larger normalized RG values than normal tissue (indicated as “+”). Figure 3(b) shows a heat map for normalized RG values higher than the threshold, whereas those below the threshold are shown in gray. An overlay of the grayscale white light image and the threshold map representing pixels with normalized RG values >1.46 (red) is displayed in Fig. 3(c). Tissue folds and pin sites showed artifacts in the normalized RG map and are indicated by arrows in Figs. 3(a) and 3(b) and were manually excluded from analyses as shown in Fig. 3(c).

### 3.2 *In vivo* imaging by WF and NLOM

**Fig. 4:**
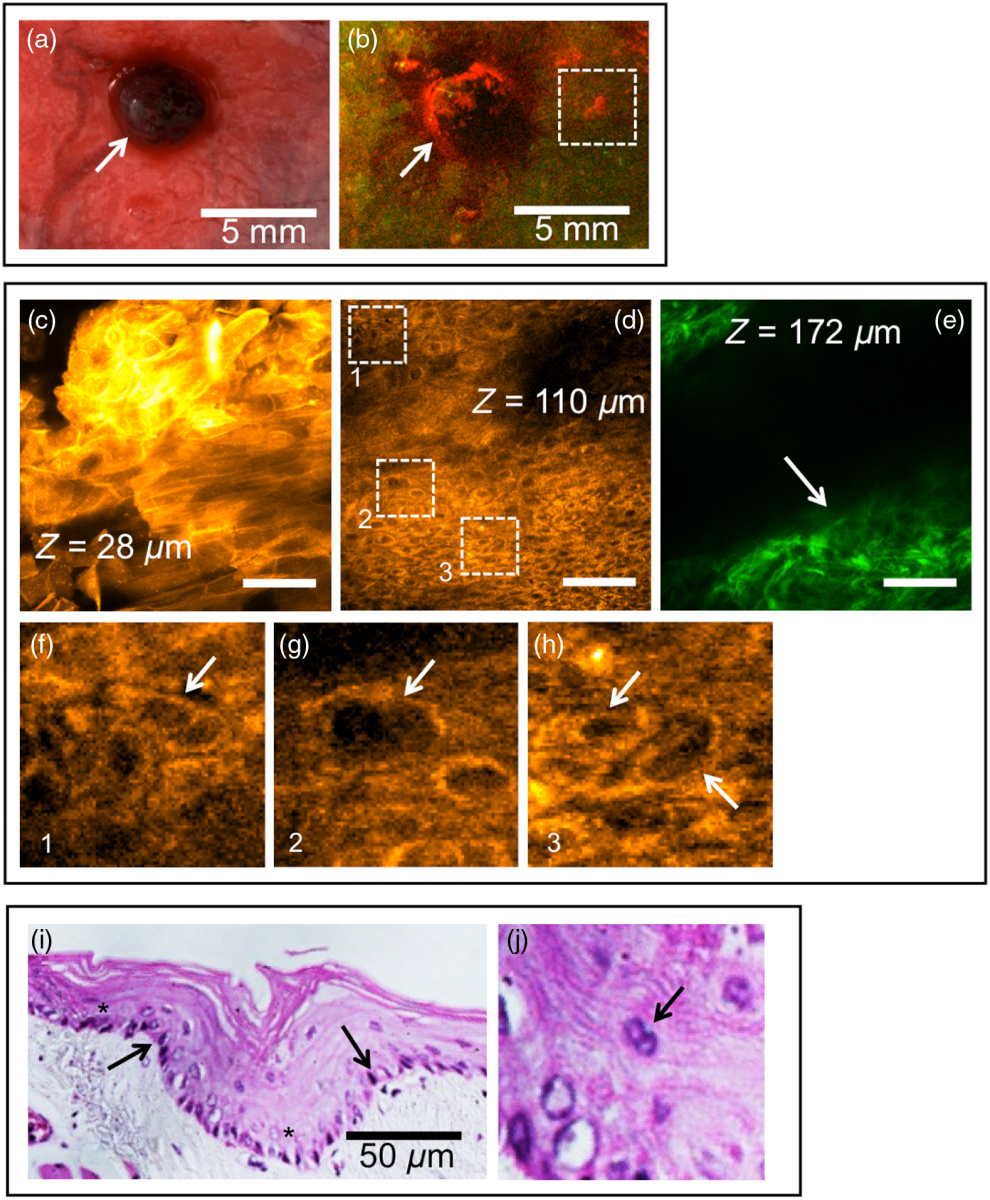
*In vivo* WF and NLOM. Multimodal imaging approach with sequential WF imaging and *in vivo* NLOM: (a) white light image and (b) WF image of areas encompassing an exophytic tumor with surrounding areas having high red and low green fluorescence. NLOM of the region of interest shown by white dotted box in (b) is displayed layer by layer as (c) keratinizing layer, (d) epithelium, and (e) ECM. White dotted boxes numbered 1, 2, and 3 in (d) are displayed in high magnification in (f), (g), and (h), respectively. Arrows point toward specific cytologic atypia: (i) H&E stained section of the tissue biopsied from the white dotted box in (b); and (j) high magnification H&E image form (j) showing multinucleated cells. Scale bars in (c), (d), and (e): 50  μm.

The multimodal imaging approach with sequential *in vivo* WF imaging and *in vivo* NLOM is described in Fig. 4. A white light image of an advanced OSCC is shown in Fig. 4(a) (indicated by “→”), which showed strong red autofluorescence [Fig. 4(b)] in WF imaging, though some areas within the tumor were dark. Apart from the tumor, several smaller areas showed increased red autofluorescence [e.g., white dotted box in Fig. 4(b)] where no visible surface abnormalities were observed in the white light image. These areas were found to harbor neoplasia and one such area is displayed in panels Figs. 4(c)–4(j) with *in vivo* layer resolved NLOM and corresponding histopathology. Representative NLOM micrographs with cytologic and architectural abnormalities, from the keratinizing layer, epithelium, and ECM are shown in Figs. 4(c)–4(e), respectively. The keratinizing layer [Fig. 4(c)] showed dyskeratosis (loss of typical honeycomb structure of keratocytes), the epithelium showed cellular atypia such as cells with enlarged nuclei and deformed nucleus and cytoplasm [Fig. 4(d)]. The ECM was vastly remodeled with loss of collagen and reduced definition of the fibers [Fig. 4(e), “→”]. Specific examples of cellular atypia such as multinucleated cells [Fig. 4(f)], enlarged nuclei [Fig. 4(g)], and anisonucleosis [nuclei with different sizes, Fig. 4(h)] are evident. Neoplastic features observed in layer-resolved NLOM were consistent with histopathology [Figs. 4(i) and 4(j)]. Biopsy and histopathologic assessment of the imaged area revealed the presence of moderate OED displaying features of neoplasia such as focal increase in epithelial thickness [Fig. 4(i), “→”], hyperchromatic nuclei [Fig. 4(i), “*”], and multinucleated cells [Fig. 4(j), “→”].

**Fig. 5:**
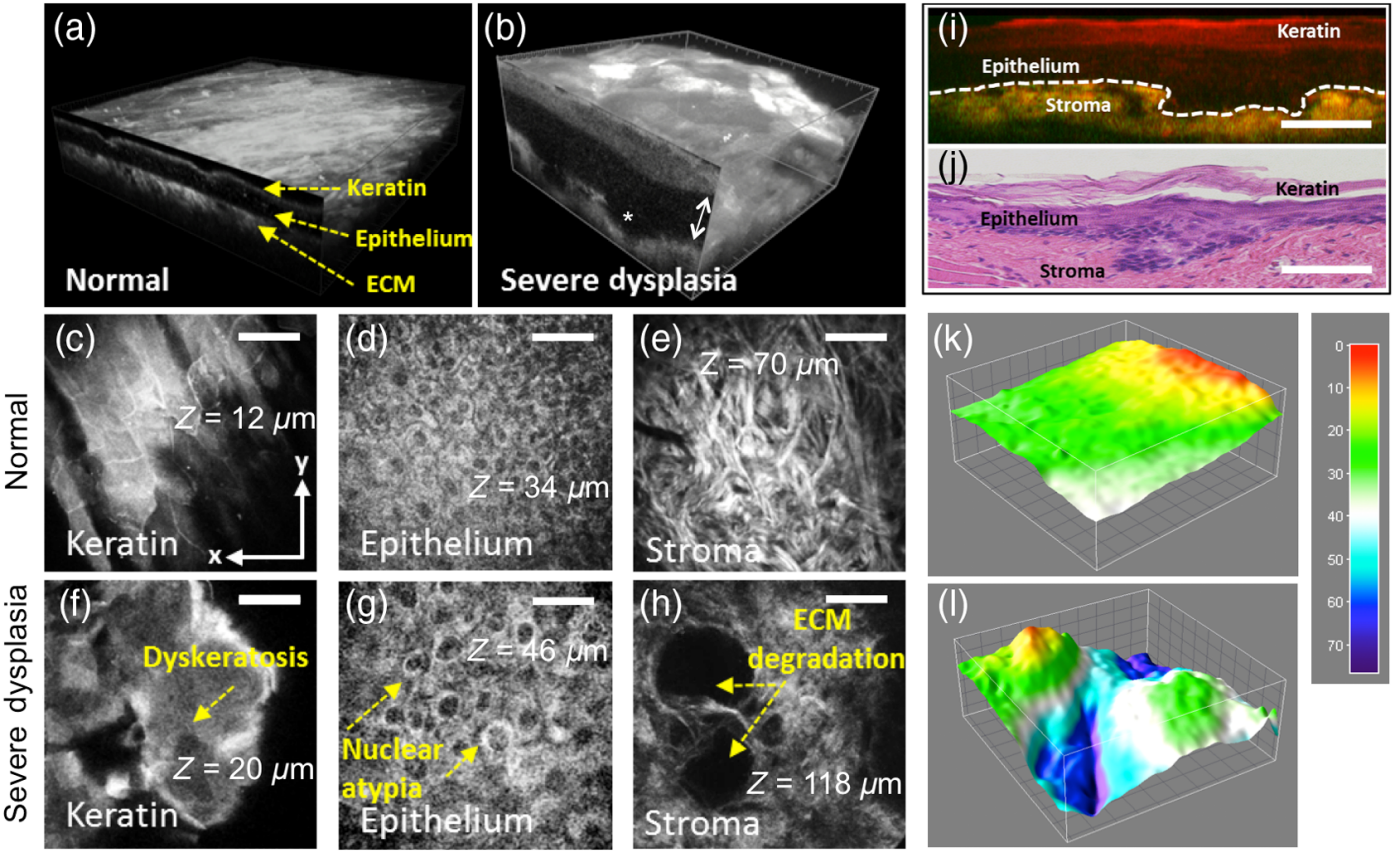
*In vivo* NLOM of hamster buccal mucosa: (a) volume reconstructions of normal and (b) severe dysplasia showing all three tissue layers (keratin, epithelium, and stroma); single optical sections of a (c) normal keratinizing layer, (d) epithelial cells, and (e) stroma are compared with (f) keratin, (g) epithelial cells, and (h) stroma of a severe dysplasia. Images shows neoplastic features such as dyskeratosis, enlarged nuclei, and ECM degradation in severe dysplasia; *In vivo* cross section of the same severe dysplasia site is shown (i) with corresponding H&E (j). ECTI surface maps generated from SHGM z stacks for normal (k) and OED (l) show changes in surface topography. The color scale next to (k) indicate depth of the ECTI surface map with red representing superficial depths and blue deepest in the surface map. Scale bars in (c)–(h): 50  μm and scale bars in (i) and (j): 40  μm.

We describe cytologic and microstructural features of normal and severe dysplasia from *in vivo* label-free NLOM and correlate with *ex vivo* histopathology in Fig. 5. Three distinct layers (keratinizing stratum corneum, epithelium, and ECM) can be observed in the three-dimensional volume reconstructions of normal [Fig. 5(a)] and severe dysplasia [Fig. 5(b)]. Signal intensity in the epithelial cells are typically lower than the autofluorescence from keratinizing layer and SHG signal from ECM, which makes the epithelial layer in the volume reconstructions appear as a dark gap between the keratin and ECM layers. The very thin keratinizing and epithelial layers as well as the flat nature of the ECM, which is seen in a normal mucosa, were disrupted in OED. Increased gap between the stratum corneum and ECM layers in severe dysplasia [Fig. 5(b), double headed arrow] indicate increased epithelial thickness. Uneven expansion of the epithelium is seen in severe dysplasia shown in Fig. 5(b) (marked “*”). Along with the epithelium, the topmost layer of stratum corneum also showed increased thickness. Single optical sections of normal stratum corneum [Fig. 5(c)], epithelium [Fig. 5(d)], and ECM [Fig. 5(e)] layers are shown in comparison to stratum corneum [Fig. 5(f)], epithelium [Fig. 5(g)], and ECM [Fig. 5(h)] layers from severe dysplasia. In OED, loss of typical honeycomb structure of keratinocytes [Fig. 5(f); “arrow”], enlarged cell nuclei [Fig. 5(g); “arrows”], and degradation of collagen fibers in the ECM [Fig. 5(h); “arrows”], all well known signatures of OED, are seen in individual optical sections. ECM of the normal epithelium [Fig. 5(e)] exhibited well-defined and thick collagen fibers, and the severe dysplasia showed loss of definition of collagen fibers and diffuse SHG signal. Cross-sectional views of an OED region from *in vivo* NLOM [Fig. 5(i)] with corresponding *ex vivo* histopathology [Fig. 5(j)] are shown for comparison. Autofluorescence signal from all three layers are displayed in “red” and SHG signal arising only from the ECM is shown in “green.” The white dotted line in Fig. 5(i) represents ECTI, which is an important site for initiation of tumor invasion. Architectural similarities between *in vivo* NLOM and histopathology were seen in all three layers such as increased thickness of keratinizing and epithelial layers and focal expansion of epithelium pushing the ECTI boundary.

ECTI surfaces (ECTI contour) for normal [Fig. 5(k)] and OED [Fig. 5(l)] regions, mapped in three-dimensions from SHGM z stacks as described in a previous study,^36^ indicate the ECM deformations created by an expanding epithelium in OED. A surface with a larger range of colors indicates more irregularities than one showing only few colors. Uneven ECTI surface in OED is clearly seen in contrast to a normal ECTI, which lacks surface irregularities.

### 3.3 *In vivo* NLOM and Image Feature Analysis

**Fig. 6:**
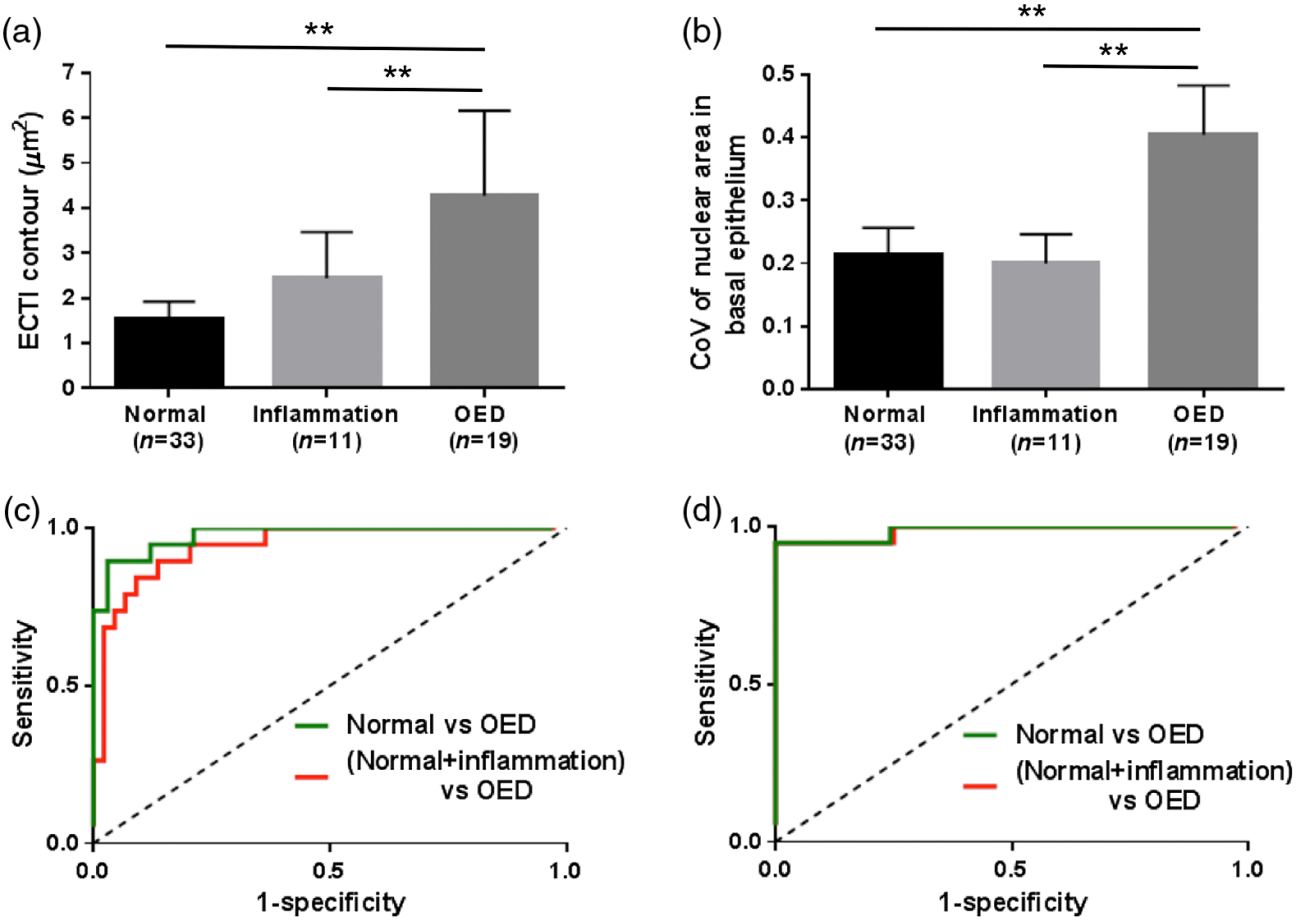
NLOM-based features of neoplasia: (a) ECTI contour and (b) coefficient of variation (CoVa) of basal nuclear area in normal, inflammation, and OED are shown. ROC curves for these parameters are shown in (c) and (d), respectively. Red and green ROC curves are for data sets in the presence and absence of inflammation. “**” represent p<0.01.

NLOM imaging results are depicted in Fig. 6 (part 2a analysis). ECTI contour [Fig. 6(a)], a measure of ECM remodeling and focal expansion of the epithelium, in normal and inflamed mucosa was relatively flat with average ECTI contour 1.55±0.37 and 2.43±1.03, respectively. ECTI from OED showed an elevated ECTI contour (4.28±1.88, p<0.01). CoVa in basal nuclear area for normal and inflammation were 0.21±0.04 and 0.2±0.05, respectively [Fig. 6(b)]. CoVa of OEDs (0.4±0.09) was significantly higher than normal and inflammation in the basal epithelium. Figures 6(c) and 6(d) show ROC curves for ECTI contour and CoVa, respectively, comparing normal and OED in the absence and presence of inflammation. ROC curves of ECTI contour showed inflammation had no effect on sensitivity (89.5%) and a minor drop in specificity from 90.9% without inflammation [Fig. 6(c), green curve] to 86.4% with inflammation [Fig. 6(c), red curve]. Similarly, while sensitivity of CoVa (94.7%) remained unchanged, specificity reduced from 100% without inflammation [Fig. 6(d), green curve] to 97.7% with inflammation [Fig. 6(d), red curve]. It is critical to note that inclusion of inflammation in the normal group reduced specificity of WF imaging to below optimal (64.15%), but inflammation had little effect on specificity of NLOM-based features of OED.

### 3.4 Image Feature Analysis with Generalized Linear Models

**Fig. 7:**
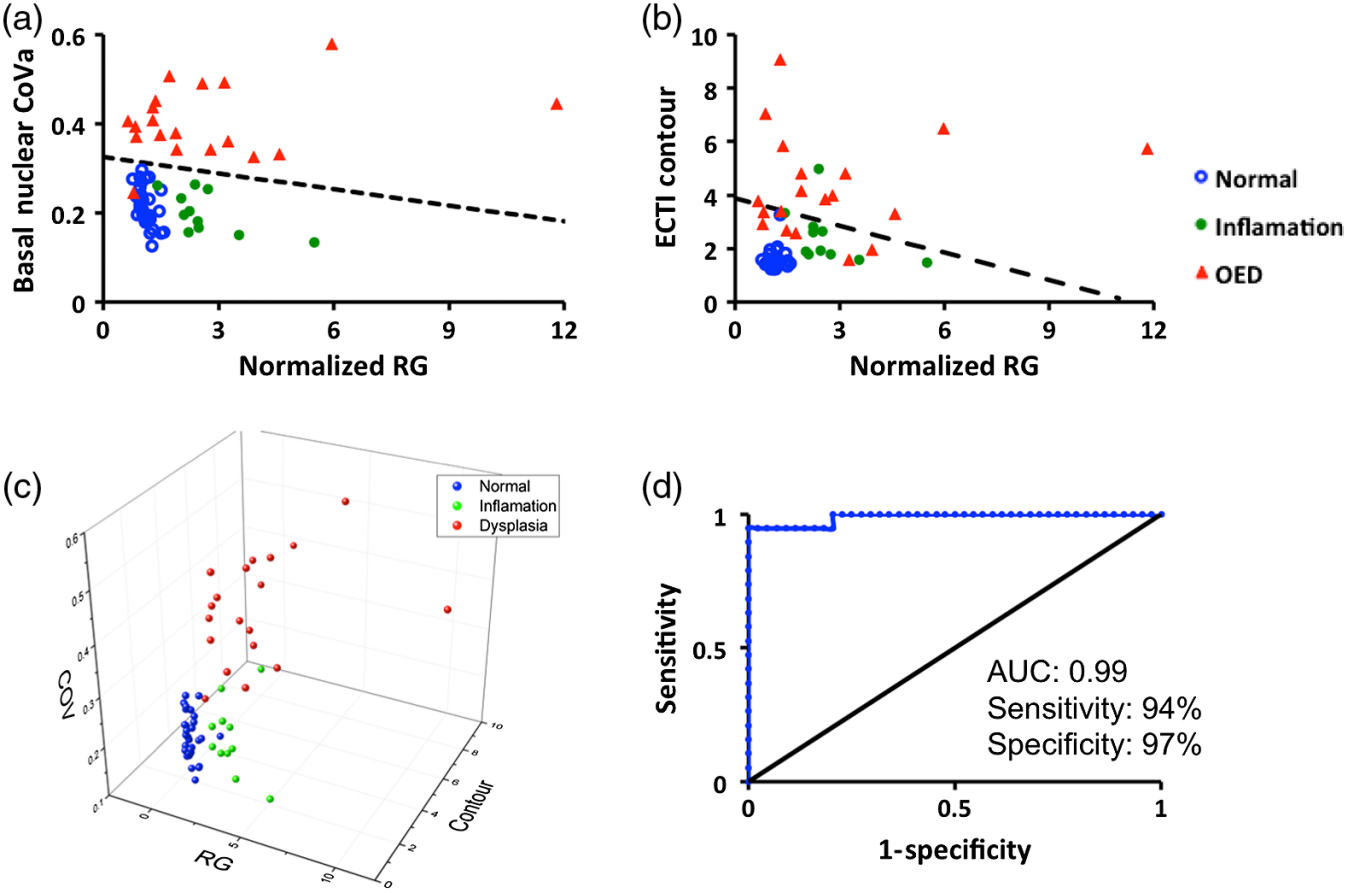
Image feature analysis with linear models: two-dimensional scatter plots for (a) normalized RG-basal nuclear CoVa and (b) normalized RG-ECTI contour show the distribution of normal, inflammation, and OEDs. The GLMs for both cases are shown as a black dotted line in the respective scatter plots; (c) a three-dimensional scatter plot with all three image-based features (normalized RG, basal nuclear CoVa, and ECTI contour); and (d) ROC curve for the three image-based features obtained from a multivariable model using forward selection method; blue: normal, green: inflammation, red: OED.

Statistical models obtained from the multimodal imaging approach to classify OEDs from non-neoplastic (normal and inflammation) tissue (part 2b) are described in Fig. 7. GLMs for normalized RG-ECTI contour [Fig. 7(a)] and normalized RG-Basal nuclear CoVa [Fig. 7(b)] demonstrated convincing discriminating power to identify OEDs. In the first model [Fig. 7(a), black dotted line], 1 out of 63 sites was misclassified, whereas in the second model [Fig. 6(b)], black dotted line], 8 out of 63 sites were misclassified. The only misclassified site in the first model was a mild OED classified as normal. Among the eight misclassified sites in the second model, seven were OEDs classified as normal, and one was inflammation classified as OED. In the pursuit of testing more robust algorithms using all three image-based parameters, a multivariable model using forward selection was developed. A scatter plot with three variables is shown in Fig. 7(c). A multivariable statistical model with normalized RG, ECTI contour, and basal nuclear CoVa was found significant (p value=0.019). After 10 iterations of parameter estimation, 98.4% sites were correctly identified. Seven OED sites out of 19 (∼37%) that showed normalized RG lower than the threshold of 1.46 (obtained from ROC analysis in Fig. 2) were correctly classified as OED in the multivariable model. ROC curve analysis of the model showed an AUC of 0.99 with 94% sensitivity and 97% specificity [Fig. 7(d)].

## 4 Discussion

This study examined feasibility of multimodal and multiscale imaging for detection of epithelial neoplasia by macroscopic WF autofluorescence imaging and NLOM microscopy by the contrasts of MPM/SHMG, tested in a preclinical model for oral neoplasia. Macroscopic WF imaging was employed to provide a large area assessment of the mucosal surface and identify areas having altered autofluorescence relative to the background as an indication of potential neoplasia. MPM/SHGM (microscopy) with limited lateral field-of-view provided subcellular resolution optical biopsy to identify subsurface microstructural features for confirmation of atypia associated with neoplasia. The choice of WF imaging with NLOM in this work was motivated by the fact that WF imaging is already a clinical, FDA-approved approach for oral cancer screening adjunct to COEs and has relatively high sensitivity; MPM/SHGM has been shown in the previous studies to have high sensitivity and specificity in preclinical tests and can provide cellular and extracellular atypia metrics.^36^ This point is important as oral neoplasia originates in the epithelium and NLOM reveals microscopic features akin to histology. This study included analyses of image metrics derived from each individual modality and included a multimodal substudy that incorporated the methods sequentially.

Though clinical WF systems exist, there is little consensus in the literature regarding the choice of optimum excitation and emission parameters for early detection efforts.^20^ Studies have indicated a multichannel approach may be superior to that of single channel green emission in clinical systems.^18^^,^^34^ Fluorescence emission in the red and green spectral windows with 405-nm excitation (normalized RG) were evaluated in this study as previous studies have shown superior performance than single-channel approaches.^29^^,^^34^^,^^71^ Since such studies had been performed in human neoplasia, it was necessary to establish WF metrics in the hamster model of oral neoplasia used here and using the setup of Fig. 1. Results indicated an increased normalized RG in the neoplastic regions that included visible tumors, small malignant lesions, and regions that under white light visualization showed no abnormality but were found through histopathology to harbor OED. However, additional sites with increased normalized RG were also present that showed no signs of neoplasia in histopathology. High sensitivity and specificity were achieved when assessments were restricted to groups of normal and neoplasia (AUC: 0.88, sensitivity: 89.83, and specificity: 83.78), but specificity decreased significantly when an SLS inflammation group (high “normalized RG”) was included together with normal (AUC: 0.83, sensitivity: 83.05, and specificity: 64.15). This trend is consistent with clinical studies that show high false positive rates when benign lesions (inflammation) are included.^19^^,^^71^^,^^72^ Such false positives result because alterations in endogenous fluorescence are not specific to neoplasia and occur with inflammation and other benign conditions.^19^^,^^21^^,^^66^ Architectural changes also impact fluorescence intensity (e.g., keratosis/increase epithelial thickness decreases “green” autofluorescence arising from the ECM). In this study, we note spectral overlap, common between red and green channels, would be mitigated in the normalized RG ratio but could possibly affect green only and red only autofluorescence assessments. Future studies could collect spectra in a widefield setup to more closely examine the effect of crosstalk that is common in filter-based and Bayer mask setups, however, as indicated above, other studies have shown multichannel WF may be superior to single (green or red) channel WF for detection.^18^^,^^34^

A novelty of this study is the inclusion of an inflammation group, as preclinical studies evaluating optical methods typically include only neoplasia versus normal controls. Part 1 of the study helped establish an RG cutoff value, above which tissues were considered potentially abnormal mucosa and visual maps highlighting such areas were explored. A normalized RG map of an entire mucosal epithelium [Fig. 3(a)] can be visualized as a heat map of potentially abnormal sites [Fig. 3(b)], to guide areas for NLOM. It is noted here that the heat map of normalized RG values does not indicate a probability of neoplasia since WF imaging showed poor specificity in the presence of inflammation. The animals were under anesthesia during measurements thus algorithms for motion correction between white light and WF were not required. Advanced algorithms to co-register WF images with the white light may be necessary in practice if these methods are translated into handheld units.

We also examined the feasibility of a sequential multimodal imaging approach, using WF with NLOM (workflow shown in Fig. 1). Metrics for analysis were guided by the studies in part 1, and a previous systematic study in the buccal mucosa that established MPM/SHGM parameters for evaluation of inflammation and neoplasia. *In vivo* NLOM at sites selected from macroscopic imaging revealed whether cellular and structural atypia were present (e.g., Figs. 4 and 5); findings shown to be in high agreement with histology in the previous studies and also in the final assessment of NLOM in the current analyses. Sites from the cheek pouch mucosa were chosen based on WF image visualization and included areas that in processing were shown to include those with normalized RG values above and below the threshold (study part 2a). The advantage of this was that it gave the opportunity to show that NLOM may provide additional knowledge to WF, confirming whether microscopic abnormalities exist, and it allowed full sensitivity/specificity assessment of final NLOM imaging. The performance of NLOM may be impacted by WF input, though the extent would depend on site selection criteria. Findings by ROC evaluation indicated outcomes consistent with previously published results, in which the mucosa was imaged only by NLOM,^36^ a consequence of selection of additional sites that did not have increased normalized RG values above the threshold. As a workflow, this sequence allows focus on areas with guidance by WF but also accounts for the possibility that additional areas can be probed using human input, considered a probable case if these methods were used in practice. An alternative automated approach would rely solely on WF to guide microscopy by large RG values and ignore low RG valued areas. Further study would be needed to evaluate outcomes on such an automated protocol with no human input and that would inherently rely on the performance of WF imaging. It is expected that in such a case, NLOM guided only by large normalized RG values would rule out false positives flagged by WF and improve overall specificity but performance would rely on WF sensitivity.

Study part 2b evaluated combined metrics from both imaging modalities with multivariable analyses (Fig. 7) could represent a different multimodal workflow, in which input from both modalities is acquired and analyzed together. The forward selection model incorporating WF with NLOM parameters together showed effective discrimination between neoplastic from benign (normal + inflammation) tissue with AUC of 0.99 (3 parameters), AUC=0.98 (GLM for normalized RG-basal nuclear CoVa), and AUC=0.95 (GLM of normalized RG-ECTI contour. These AUCs were greater than in the prior analysis of normalized RG alone (AUC=0.83, Fig. 2, Table 1) and ECTI contour (AUC=0.91, Fig. 6). Basal nuclear CoVa alone (AUC=0.99, Fig. 6) appeared to perform as good as the multivariable model. However, without a prescreening technique to guide NLOM, detection of suspicious areas would be challenging.

As a model, the hamster buccal model presented a heterogenous mucosal surface that harbors multiple areas of dysplasia and OSCC, similarities to human tissue histological and molecular features, and fluorescence properties as determined by the previous studies. A limitation for this study was the presence of large exophytic tumors that, while possible to image by WF, were challenging to center and image with microscopy without tissue compression and bleeding. Thus in the multimodal imaging study, tumors were not imaged by NLOM. As most human OSCC do not protrude in the same manner, this was an accepted exclusion but indicates the need for future studies of NLOM imaging in human OSCC and multimodal imaging in human OSCC. It is noted that the feasibility of NLOM imaging in human OED and OSCC have been shown previously.^57^^,^^73^

Going forward, efforts should consider how modalities might be integrated into a workflow that provides useful guidance for biopsy. Clinical WF systems are handheld devices and with the development of fiber-based NLOM one can envision scenarios, in which microscopy is guided by images from the WF device as a separate system, or into a combined system. In a recent study, a multimodal imaging approach with WF imaging to guide HRME as separate systems was presented.^56^ The study demonstrated probable workflows between separate modalities and developed automated algorithms for image-based feature extraction and classification that can possibly be applied across imaging technologies. Although HRME requires staining of the mucosa for contrast, NLOM as used in this study and CRM are label-free.^74^ Both HRME and CRM rely on single planes across the surface, though the latter has the technical capability for acquiring depth information. NLOM could offer advantages in depth imaging ability beyond 1 mm that allows assessment of cellular, ECTI, and ECM features.^75^^–^^77^ Label-free NLOM with endogenous fluorescence arising from metabolic species (e.g., NADH) could provide additional functional/molecular information (e.g., metabolic heterogeneity). The cost of NLOM is a factor that should be considered with the major costs expected to be excitation lasers in bench systems. However, low-cost fiber-lasers are now available, which could help in translation and integration, particularly for the high resource setting. Recent development of endoscopic NLOM systems establishes the feasibility of compactness, and as costs continue to decrease potential translation of WF-NLOM approaches could be realized after thorough investigation and development.^52^

## 5 Conclusions

The results presented here show the feasibility of a multiscale imaging approach for combined large area assessment and subsurface NLOM of neoplastic oral mucosa. The study demonstrates how respective strengths and weaknesses of each method may be leveraged to provide large area rapid assessment with depth-resolved cytology and ECTI assessment of select areas. Multivariable statistical analyses indicate the potential for high sensitivity–specificity detection of neoplasia using metrics from both methods, particularly when metrics are analyzed together. Success of multimodal imaging approaches in preclinical studies such as presented here is expected to motivate future studies with improved WF parameters and application in human OSCC, and specifically in the case of WF and NLOM, future studies should focus on evaluations in human OSCC.

## References

[1] American Cancer Society, “Key statistics for oral cavity and oropharyngeal cancers,” 2020, https://www.cancer.org/cancer/oral-cavity-and-oropharyngeal-cancer/about/key-statistics.html (updated 8 January 2020; cited 14 April 2020).

[2] National Institute of Health, “Cancer stat facts: oral cavity and pharynx cancer,” 2020, https://seer.cancer.gov/statfacts/html/oralcav.html (cited April 2020).

[3] Bezerra N. V. et al., “Impact of the anatomical location, alcoholism and smoking on the prevalence of advanced oral cancer in Brazil,” Med. Oral Patol. Oral Cir. Bucal. 23(3), e295–e301 (2018). 10.4317/medoral.22318 29680854 PMC5945237

[4] Güneri P Epstein J. B., “Late stage diagnosis of oral cancer: components and possible solutions,” Oral Oncol. 50(12), 1131–1136 (2014). EJCCER 1368-8375 10.1016/j.oraloncology.2014.09.005 25255960

[5] Howlader N. et al., “SEER Cancer Statistics Review, 1975–2016,” National Cancer Institute, 1975-2016, https://seer.cancer.gov/csr/1975_2016/ (updated April 9, 2020).

[6] Slootweg P. J. El-Naggar A. K., “World Health Organization 4th edition of head and neck tumor classification: insight into the consequential modifications,” Virchows Arch. 472(3), 311–313 (2018). 10.1007/s00428-018-2320-6 29450648

[7] Soares A. B. Perschbacher K. Perez-Ordonez B., “Oral potentially malignant disorders,” Diagn. Histopathol. 24(5), 161–165 (2018). DIHIDH 0272-7749 10.1016/j.mpdhp.2018.03.005

[8] Ganesh D. et al., “Potentially malignant oral disorders and cancer transformation,” Anticancer Res. 38(6), 3223–3229 (2018). ANTRD4 0250-7005 10.21873/anticanres.12587 29848669

[9] Warnakulasuriya S., “Clinical features and presentation of oral potentially malignant disorders,” Oral Surg. Oral Med. Oral Pathol. Oral Radiol. 125(6), 582–590 (2018). 10.1016/j.oooo.2018.03.011 29673799

[10] Lingen M. W. et al., “Critical evaluation of diagnostic aids for the detection of oral cancer,” Oral Oncol. 44(1), 10–22 (2008). EJCCER 1368-8375 10.1016/j.oraloncology.2007.06.011 17825602 PMC2424250

[11] Epstein J. B. et al., “Screening for and diagnosis of oral premalignant lesions and oropharyngeal squamous cell carcinoma: role of primary care physicians,” Can. Fam. Physician 54(6), 870–875 (2008). 18556495 PMC2426981

[r12] Lim K. et al., “Opportunistic screening for oral cancer and precancer in general dental practice: results of a demonstration study,” Br. Dent. J. 194(9), 497–502; discussion 493 (2003). 10.1038/sj.bdj.4810069 12835785

[13] Mathew B. et al., “Evaluation of mouth self-examination in the control of oral cancer,” Br. J. Cancer 71(2), 397–399 (1995). BJCAAI 0007-0920 10.1038/bjc.1995.81 7841060 PMC2033573

[14] Gray L. et al., “Perceptions of tongue lesions by dental hygiene students and otolaryngologists,” J. Cancer Educ. 17(4), 191–195 (2002). 10.1080/08858190209528836 12556054

[15] Carreras-Torras C. Gay-Escoda C., “Techniques for early diagnosis of oral squamous cell carcinoma: systematic review,” Med Oral Patol Oral Cir Bucal. 20(3), e305–e315 (2015). 10.4317/medoral.20347 25662554 PMC4464918

[16] Epstein J. B. et al., “The limitations of the clinical oral examination in detecting dysplastic oral lesions and oral squamous cell carcinoma,” J. Am. Dent. Assoc. 143(12), 1332–1342 (2012). 10.14219/jada.archive.2012.0096 23204089

[17] De Veld D. C. et al., “The status of in vivo autofluorescence spectroscopy and imaging for oral oncology,” Oral Oncol. 41(2), 117–131 (2005). EJCCER 1368-8375 10.1016/j.oraloncology.2004.07.007 15695112

[r18] Roblyer D. et al., “Multispectral optical imaging device for in vivo detection of oral neoplasia,” J. Biomed. Opt. 13(2), 024019 (2008). JBOPFO 1083-3668 10.1117/1.2904658 18465982 PMC3970814

[19] Shin D. et al., “Advances in fluorescence imaging techniques to detect oral cancer and its precursors,” Future Oncol. 6(7), 1143–1154 (2010). 10.2217/fon.10.79 20624126 PMC2929485

[20] Ramanujam N., “Fluorescence spectroscopy of neoplastic and non-neoplastic tissues,” Neoplasia 2(1–2), 89–117 (2000). 10.1038/sj.neo.7900077 10933071 PMC1531869

[21] Pavlova I. et al., “Understanding the biological basis of autofluorescence imaging for oral cancer detection: high-resolution fluorescence microscopy in viable tissue,” Clin. Cancer Res. 14(8), 2396–2404 (2008). 10.1158/1078-0432.CCR-07-1609 18413830 PMC2773159

[22] de Veld D. C. et al., “Autofluorescence and Raman microspectroscopy of tissue sections of oral lesions,” Lasers Med. Sci. 19(4), 203–209 (2005). 10.1007/s10103-004-0325-7 15772873

[23] Mehrotra R. et al., “A cross-sectional study evaluating chemiluminescence and autofluorescence in the detection of clinically innocuous precancerous and cancerous oral lesions,” J. Am. Dent. Assoc. 141(2), 151–156 (2010). 10.14219/jada.archive.2010.0132 20123872

[24] Sambandham T. et al., “The application of vizilite in oral cancer,” J. Clin. Diagn. Res. 7(1), 185–186 (2013). 10.7860/JCDR/2012/5163.2704 23450083 PMC3576785

[25] Poh C. F. et al., “Fluorescence visualization detection of field alterations in tumor margins of oral cancer patients,” Clin. Cancer Res. 12(22), 6716–6722 (2006). 10.1158/1078-0432.CCR-06-1317 17121891

[26] Lane P. M. et al., “Simple device for the direct visualization of oral-cavity tissue fluorescence,” J. Biomed. Opt. 11(2), 024006 (2006). JBOPFO 1083-3668 10.1117/1.2193157 16674196

[27] Farah C. S. et al., “Efficacy of tissue autofluorescence imaging (VELScope) in the visualization of oral mucosal lesions,” Head Neck 34(6), 856–862 (2012). 10.1002/hed.21834 21818819

[r28] Hanken H. et al., “The detection of oral pre-malignant lesions with an autofluorescence based imaging system (VELscope)—a single blinded clinical evaluation,” Head Face Med. 9, 23 (2013). 10.1186/1746-160X-9-23 23967796 PMC3851797

[r29] Koch F. P. et al., “Effectiveness of autofluorescence to identify suspicious oral lesions--a prospective, blinded clinical trial,” Clin. Oral Invest. 15(6), 975–982 (2011). 10.1007/s00784-010-0455-1 20714910

[r30] Awan K. H. Morgan P. R. Warnakulasuriya S., “Evaluation of an autofluorescence based imaging system (VELscope) in the detection of oral potentially malignant disorders and benign Keratoses,” Oral Oncol. 47(4), 274–277 (2011). EJCCER 1368-8375 10.1016/j.oraloncology.2011.02.001 21396880

[r31] Amirchaghmaghi M. et al., “The diagnostic value of the native fluorescence visualization device for early detection of premalignant/malignant lesions of the oral cavity,” Photodiagn. Photodyn. Ther. 21, 19–27 (2018). 10.1016/j.pdpdt.2017.10.019 29079347

[r32] Scheer M. et al., “Autofluorescence imaging in recurrent oral squamous cell carcinoma,” Oral Maxillofac. Surg. 20(1), 27–33 (2016). 10.1007/s10006-015-0520-7 26267490

[33] Mascitti M. et al., “An overview on current non-invasive diagnostic devices in oral oncology,” Front. Physiol. 9, 1510 (2018). FROPBK 0301-536X 10.3389/fphys.2018.01510 30410451 PMC6209963

[r34] Roblyer D. et al., “Objective detection and delineation of oral neoplasia using autofluorescence imaging,” Cancer Prev. Res. 2(5), 423–431 (2009). 10.1158/1940-6207.CAPR-08-0229 PMC2719708 19401530

[35] Rollakanti K. R. et al., “Techniques for fluorescence detection of protoporphyrin IX in skin cancers associated with photodynamic therapy,” Photonics Lasers Med. 2(4), 287–303 (2013). 10.1515/plm-2013-0030 25599015 PMC4295789

[36] Pal R. et al., “Remodeling of the epithelial-connective tissue interface in oral epithelial dysplasia as visualized by noninvasive 3D imaging,” Cancer Res. 76(16), 4637–4647 (2016). CNREA8 0008-5472 10.1158/0008-5472.CAN-16-0252 27302162 PMC4987238

[37] Skala M. C. et al., “Multiphoton microscopy of endogenous fluorescence differentiates normal, precancerous, and cancerous squamous epithelial tissues,” Cancer Res. 65(4), 1180–1186 (2005). CNREA8 0008-5472 10.1158/0008-5472.CAN-04-3031 15735001 PMC4189807

[38] Davies K. et al., “Point of care optical diagnostic technologies for the detection of oral and oropharyngeal squamous cell carcinoma,” Surgeon 13(6), 321–329 (2015). 10.1016/j.surge.2015.06.004 26148762

[39] Rosenthal E. L. et al., “The status of contemporary image-guided modalities in oncologic surgery,” Ann. Surg. 261(1), 46–55 (2015). 10.1097/SLA.0000000000000622 25599326 PMC4299947

[40] Yoon Y. et al., “In vivo wide-field reflectance/fluorescence imaging and polarization-sensitive optical coherence tomography of human oral cavity with a forward-viewing probe,” Biomed. Opt. Express 6(2), 524–535 (2015). BOEICL 2156-7085 10.1364/BOE.6.000524 25780742 PMC4354576

[41] Stasio D. D. et al., “Measurement of oral epithelial thickness by optical coherence tomography,” Diagnostics 9(3), 90 (2019). 10.3390/diagnostics9030090 PMC6787684 31390841

[42] Edward K. et al., “In vivo layer-resolved characterization of oral dysplasia via nonlinear optical micro-spectroscopy,” Biomed. Opt. Express 3(7), 1579–93 (2012). BOEICL 2156-7085 10.1364/BOE.3.001579 22808430 PMC3395483

[r43] Levitt J. M. et al., “Automated biochemical, morphological, and organizational assessment of precancerous changes from endogenous two-photon fluorescence images,” PLoS One 6(9), e24765 (2011). POLNCL 1932-6203 10.1371/journal.pone.0024765 21931846 PMC3170385

[r44] Skala M. C. et al., “In vivo multiphoton microscopy of NADH and FAD redox states, fluorescence lifetimes, and cellular morphology in precancerous epithelia,” Proc. Natl. Acad. Sci. U. S. A. 104(49), 19494 (2007). PNASA6 0027-8424 10.1073/pnas.0708425104 18042710 PMC2148317

[r45] Walsh A. J. et al., “Ex vivo optical metabolic measurements from cultured tissue reflect in vivo tissue status,” J. Biomed. Opt. 17(11), 116015 (2012). JBOPFO 1083-3668 10.1117/1.JBO.17.11.116015 23117810 PMC3484268

[r46] Pal R. et al., “Spectroscopic characterization of oral epithelial dysplasia and squamous cell carcinoma using multiphoton autofluorescence micro-spectroscopy,” Lasers Surg. Med. 49(9), 866–873 (2017). LSMEDI 0196-8092 10.1002/lsm.22697 28677822 PMC5643238

[r47] Pal R. et al., “In-vivo nonlinear optical microscopy (NLOM) of epithelial-connective tissue interface (ECTI) reveals quantitative measures of neoplasia in hamster oral mucosa,” PLoS One 10(1), e0116754 (2015). POLNCL 1932-6203 10.1371/journal.pone.0116754 25633927 PMC4310593

[r48] Vargas G. et al., “Multiphoton autofluorescence microscopy and second harmonic generation microscopy of oral epithelial neoplasms,” in Annu. Int. Conf. IEEE Eng. Med. and Biol. Soc., pp. 6311–6313 (2009). 10.1109/IEMBS.2009.5332783 PMC2829879 19963923

[r49] Wilder-Smith P. et al., “In vivo multiphoton fluorescence imaging: a novel approach to oral malignancy,” Lasers Surg. Med. 35(2), 96–103 (2004). LSMEDI 0196-8092 10.1002/lsm.20079 15334611

[50] Wilder-Smith P. et al., “Noninvasive imaging of oral premalignancy and malignancy,” J. Biomed. Opt. 10(5), 051601 (2005). JBOPFO 1083-3668 10.1117/1.2098930 16292949

[51] Shah T. A. Melissa C. S., “Ex vivo label-free microscopy of head and neck cancer patient tissues,” Proc. SPIE 9329, 93292B (2015). PSISDG 0277-786X 10.1117/12.2075583

[52] Li A. et al., “A biopsy-needle compatible varifocal multiphoton rigid probe for depth-resolved optical biopsy,” J. Biophotonics 12(1), e201800229 (2019). 10.1002/jbio.201800229 30117286 PMC6325015

[r53] Rivera D. R. et al., “Compact and flexible raster scanning multiphoton endoscope capable of imaging unstained tissue,” Proc. Natl. Acad. Sci. U. S. A. 108(43), 17598–17603 (2011). PNASA6 0027-8424 10.1073/pnas.1114746108 22006303 PMC3203813

[54] Zhang Y. et al., “A compact fiber-optic SHG scanning endomicroscope and its application to visualize cervical remodeling during pregnancy,” Proc. Natl. Acad. Sci. U. S. A. 109(32), 12878 (2012). PNASA6 0027-8424 10.1073/pnas.1121495109 22826263 PMC3420182

[55] Pierce M. C. et al., “Accuracy of in vivo multimodal optical imaging for detection of oral neoplasia,” Cancer Prev. Res. 5(6), 801–809 (2012). 10.1158/1940-6207.CAPR-11-0555 PMC3560936 22551901

[56] Yang E. C. et al., “Development of an integrated multimodal optical imaging system with real-time image analysis for the evaluation of oral premalignant lesions,” J. Biomed. Opt. 24(2), 025003 (2019). JBOPFO 1083-3668 10.1117/1.JBO.24.2.025003 PMC6383051 30793567

[57] Malik B. H. et al., “A novel multimodal optical imaging system for early detection of oral cancer,” Oral Surg. Oral Med. Oral Pathol. Oral Radiol. 121(3), 290–300.e2 (2016). 10.1016/j.oooo.2015.10.020 26725720 PMC4752880

[58] Jabbour J. M. et al., “Fluorescence lifetime imaging and reflectance confocal microscopy for multiscale imaging of oral precancer,” J. Biomed. Opt. 18(4), 046012 (2013). JBOPFO 1083-3668 10.1117/1.JBO.18.4.046012 23595826 PMC3628018

[59] Hucker W. J. et al., “Bimodal biophotonic imaging of the structure-function relationship in cardiac tissue,” J. Biomed. Opt. 13(5), 054012 (2008). JBOPFO 1083-3668 10.1117/1.2975826 19021392 PMC2719892

[60] Park J. et al., “A dual-modality optical coherence tomography and fluorescence lifetime imaging microscopy system for simultaneous morphological and biochemical tissue characterization,” Biomed. Opt. Express 1(1), 186–200 (2010). BOEICL 2156-7085 10.1364/BOE.1.000186 21258457 PMC3005181

[61] Gimenez-Conti I., “The hamster cheek pouch carcinogenesis model,” Acta Odontol. Latinoam. 7(1), 3–12 (1993). AOLAEN 11885256

[62] Vairaktaris E. et al., “The hamster model of sequential oral oncogenesis,” Oral Oncol. 44(4), 315–324 (2008). EJCCER 1368-8375 10.1016/j.oraloncology.2007.08.015 18061531

[63] Baert J. H. et al., “The effect of sodium lauryl sulphate and triclosan on hamster cheek pouch mucosa,” Int. J. Exp. Pathol. 77(2), 73–78 (1996). IJEPEI 1365-2613 10.1046/j.1365-2613.1996.00965.x 8762865 PMC2691625

[64] Veys R. J. Baert J. H. De Boever J. A., “Histological changes in the hamster cheek pouch epithelium induced by topical application of sodium lauryl sulphate,” Int. J. Exp. Pathol. 75(3), 203–209 (1994). IJEPEI 1365-2613 8086317 PMC2001810

[65] Müller S., “Update from the 4th Edition of the World Health Organization of head and neck tumours: tumours of the oral cavity and mobile tongue,” Head Neck Pathol. 11(1), 33–40 (2017). 10.1007/s12105-017-0792-3 28247230 PMC5340733

[66] Pal R. et al., “In-vivo topical mucosal delivery of a fluorescent deoxy-glucose delineates neoplasia from normal in a preclinical model of oral epithelial neoplasia,” Sci. Rep. 8(1), 9760 (2018). SRCEC3 2045-2322 10.1038/s41598-018-28014-8 29950704 PMC6021424

[67] Syafriadi M. et al., “Two-phase appearance of oral epithelial dysplasia resulting from focal proliferation of parabasal cells and apoptosis of prickle cells,” J. Oral Pathol. Med. 34(3), 140–149 (2005). JPMEEA 0904-2512 10.1111/j.1600-0714.2004.00283.x 15689227

[68] Tilakaratne W. M. et al., “Grading oral epithelial dysplasia: analysis of individual features,” J. Oral Pathol. Med. 40(7), 533–540 (2011). JPMEEA 0904-2512 10.1111/j.1600-0714.2011.01033.x 21501232

[69] Vulliez M. et al., “Multi-scale curvature analysis and correlations with the fatigue limit on steel surfaces after milling,” Procedia CIRP 13, 308–313 (2014). 10.1016/j.procir.2014.04.052

[70] Song L. Langfelder P. Horvath S., “Random generalized linear model: a highly accurate and interpretable ensemble predictor,” BMC Bioinf. 14(1), 5 (2013). BBMIC4 1471-2105 10.1186/1471-2105-14-5 PMC3645958 23323760

[71] Strome A. et al., “Current practice and emerging molecular imaging technologies in oral cancer screening,” Mol. Imaging 17, 1536012118808644 (2018). 10.1177/1536012118808644 32852263 PMC6287312

[72] Brocklehurst P. R. Speight P. M., “Screening for mouth cancer: the pros and cons of a national programme,” Br. Dent. J. 225(9), 815–819 (2018). 10.1038/sj.bdj.2018.918 30412550

[73] Perry S. W. Burke R. M. Brown E. B., “Two-photon and second harmonic microscopy in clinical and translational cancer research,” Ann. Biomed. Eng. 40(2), 277–291 (2012). ABMECF 0090-6964 10.1007/s10439-012-0512-9 22258888 PMC3342697

[74] Peterson G. et al., “Feasibility of a video-mosaicking approach to extend the field-of-view for reflectance confocal microscopy in the oral cavity in vivo,” Lasers Surg. Med. 51(5), 439–451 (2019). LSMEDI 0196-8092 10.1002/lsm.23090 PMC6842028 31067360

[75] Miller D. R. et al., “Deep tissue imaging with multiphoton fluorescence microscopy,” Curr. Opin. Biomed. Eng. 4, 32–39 (2017). 10.1016/j.cobme.2017.09.004 29335679 PMC5766275

[r76] Miller D. R. et al., “In vivo multiphoton microscopy beyond 1 mm in the brain,” Proc. SPIE 10069, 100690C (2017). PSISDG 0277-786X 10.1117/12.2249949

[77] Kobat D. Horton N. G. Xu C., “In vivo two-photon microscopy to 1.6-mm depth in mouse cortex,” J. Biomed. Opt. 16(10), 106014 (2011). JBOPFO 1083-3668 10.1117/1.3646209 22029361

